# How to Use Localized Surface Plasmon for Monitoring the Adsorption of Thiol Molecules on Gold Nanoparticles?

**DOI:** 10.3390/nano12020292

**Published:** 2022-01-17

**Authors:** Angeline S. Dileseigres, Yoann Prado, Olivier Pluchery

**Affiliations:** CNRS, Institut des Nanosciences de Paris (INSP) UMR7588, Sorbonne Université, 4 Place Jussieu, 75005 Paris, France; angeline.dileseigres@insp.jussieu.fr (A.S.D.); yoann.prado@insp.jussieu.fr (Y.P.)

**Keywords:** gold nanoparticles, functionalization, thiol, localized surface plasmon resonance

## Abstract

The functionalization of spherical gold nanoparticles (AuNPs) in solution with thiol molecules is essential for further developing their applications. AuNPs exhibit a clear localized surface plasmon resonance (LSPR) at 520 nm in water for 20 nm size nanoparticles, which is extremely sensitive to the local surface chemistry. In this study, we revisit the use of UV-visible spectroscopy for monitoring the LSPR peak and investigate the progressive reaction of thiol molecules on 22 nm gold nanoparticles. FTIR spectroscopy and TEM are used for confirming the nature of ligands and the nanoparticle diameter. Two thiols are studied: 11-mercaptoundecanoic acid (MUDA) and 16-mercaptohexadecanoic acid (MHDA). Surface saturation is detected after adding 20 nmol of thiols into 1.3 × 10^−3^ nmol of AuNPs, corresponding approximately to 15,000 molecules per AuNPs (which is equivalent to 10.0 molecules per nm^2^). Saturation corresponds to an LSPR shift of 2.7 nm and 3.9 nm for MUDA and MHDA, respectively. This LSPR shift is analyzed with an easy-to-use analytical model that accurately predicts the wavelength shift. The case of dodecanehtiol (DDT) where the LSPR shift is 15.6 nm is also quickly commented. An insight into the kinetics of the functionalization is obtained by monitoring the reaction for a low thiol concentration, and the reaction appears to be completed in less than one hour.

## 1. Introduction

Gold nanoparticles (AuNPs) have been established as essential nano-objects in many research areas and for many nanotechnology applications. In particular, plasmonics [1,2,3,4], biosensing [5,6,7,8], biomedicine [9,10,11], catalysis [12,13], molecular electronics [9,14,15,16], nanoelectronics [17,18,19], or spintronics [20,21] have shown that the nanoparticle surface must be properly controlled for producing the desired effects. This surface is often functionalized with a tailored molecular layer whose nature, thickness, and organization control the final properties. However, producing evidence of the surface functionalization and unravelling the functionalization processes remain a challenging issue. For example, the thermodynamic and the kinetic processes of the adsorption of thiol ligands differ on a flat gold surface and on the curved nanoparticle surface. Namely, this surface chemistry depends on the radius of curvature as well as on the arrangement of the binding sites [22]. Regarding AuNPs synthesis, the Turkevich method is probably the most popular one [23,24,25], and the nanoparticles are stabilized by a layer of citrate molecules [26,27]. A key advantage of this synthesis is that citrates are only weakly bound to the AuNP surface and can be easily replaced by most of the molecules prone to bind to gold. In particular, thiol derivatives are the molecules of choice for such ligand exchange [28,29,30]. However, there are still many open questions about the gold–ligand bond, the final surface coverage, and the time for a complete replacement. For example, the group of Stellaci demonstrated that a complete ligand exchange from citrate to 11-mercaptoundecanoate could be achieved on AuNPs. For that purpose, they design a clever sequence of cryo-TEM (Transmission Electron Microscope) images to visualize the progressive replacement of the initial anions [28]. They have shown that 2800 alkanethiolates were necessary for saturating the 14 nm nanoparticle surface. They also used UV-visible spectroscopy and the plasmon of AuNPs for accessing the kinetics. Some authors employ thermogravimetry analysis for evaluating the nature of ligands at the nanoparticle surface [31,32,33]. However, in such experiments, ligand exchange may also destabilize the nanoparticle suspension when it suppresses the electrostatic repulsions between nanoparticles [34]. It happens when the negatively charged citrates are replaced by neutral or positively charged ligands [35]. Aggregation can subsequently start either in a reversible [36] or irreversible process [37].

A very efficient approach for investigating the nanoparticle functionalization is brought by the localized surface plasmon resonance (LSPR). Actually, AuNPs in colloidal solutions are characterized by a pronounced LSPR, which is detected at a wavelength of 520 nm for spherical AuNPs of 20 nm diameter in water. LSPR allows monitoring the AuNPs functionalization thanks to the well-documented fact that the resonance wavelength shifts upwards when the refractive index of the surrounding medium increases, which is the case when molecules start adsorbing on the nanoparticle surface [38,39]. This shift of the resonance is measured straightforwardly with UV-visible spectroscopy, which is easy to handle and can be used for all types of ligands, compared to fluorescence spectroscopy, which is limited to ligands possessing a fluorophore [22,30].

**Figure 1:**
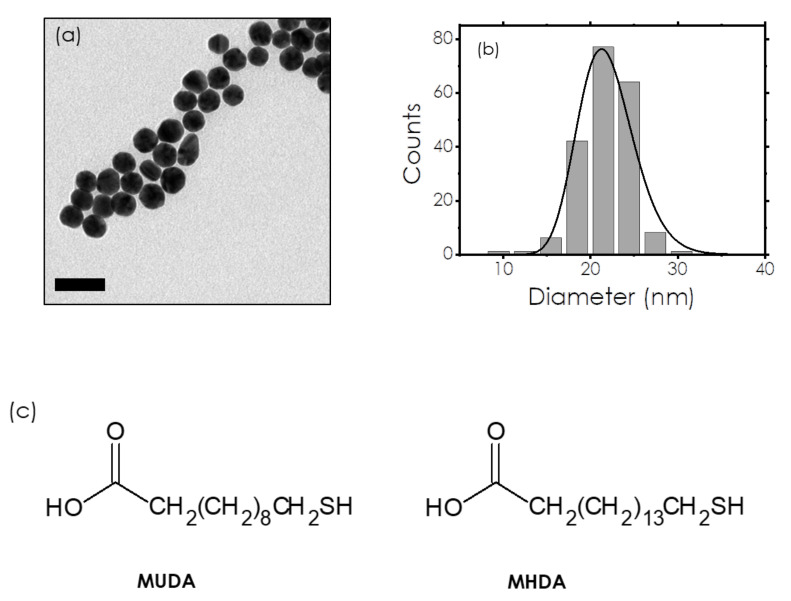
(**a**) Transmission Electronic Microscope (TEM) picture of nanoparticles stabilized with citrate prior to any thiol functionalization (scale bar 50 nm). (**b**) Size distribution of the nanoparticles and lognormal fit showing an average size of 21.7 (σ=0.15). (**c**) Chemical structures and abbreviation of the alkanethiols used in this study: 11-mercaptoundecanoic acid (MUDA) and 16-mercaptohexadecanoic acid (MHDA).

In the present study, we present the methodology for taking full advantage of the LSPR when investigating plasmonic nanoparticle functionalization. We combine experimental data where the LSPR wavelength shift is analyzed with a set of easy-to-use analytical models that make it possible to discuss quantitatively the values of this shift. Although most of the elements can be found scattered in earlier publications, such a methodology was never published in such a practical way to the best of our knowledge. Here, we illustrate our methodology with 22 nm citrate-stabilized AuNPs (citrate-AuNPs) functionalized with two different thiols, as presented in Figure 1: 11-mercaptoundecanoic acid (MUDA) and 16-mercaptohexadecanoic acid (MHDA). These molecules have been chosen because they possess an anionic charge in the deprotonated state that ensures a colloidal stability after the replacement of negatively charged citrate molecules. Nevertheless, they differ in the length of the carbon backbone that can affect differently the stability of the nanoparticles. The evolution of the LSPR peak position was monitored as well as its intensity (maximum value of the absorbance spectrum), and it is shown that the full functionalization of the nanoparticles results in LSPR wavelength redshifts of 2.7 nm (MUDA) and 3.9 nm (MHDA). These shifts are well reproduced by the analytical model we have developed and which is based on the combination of geometrical considerations and standard analytical models known in plasmonics. In addition, the methods presented in this study (experiments and calculations) can be generalized to a large variety of functionalizing molecules and used for other plasmonic nanoparticles.

## 2. Materials and Methods

**Materials.** Hydrogen tetrachloroaurate(III) hydrate (HAuCl_4_·H_2_O, Sigma-Aldrich, St. Louis, MO, USA, 50% Au basis), sodium citrate dihydrate (SC, Fluka, >99.0%), 11-mercaptoundecanoic acid (MUDA, Sigma-Aldrich, 98%), 16-mercaptohexadecanoic acid (MHDA, Sigma-Aldrich, 90%), and ethanol absolute (VWR, Normapur, Radnor, PA, USA) were used as received. Deionized water (18 MΩ) was used in all experiments. Glassware was clean with *aqua regia*; then, it was repeatedly rinsed with demineralized water and let in a water bath overnight. Caution: Aqua regia is highly toxic and corrosive; it requires personal protective equipment and should be handled under a fume hood. Float-zone (FZ) silicon substrates Si(100) were purchased from Neyco (Vanves, France). Prior to any treatments, the silicon pieces were cleaned under sonication with acetone then with ethanol.

**AuNPs synthesis.** First, 22 nm AuNPs were synthesized following a modified Turkevich–Frens protocol [24,40]. In a 100 mL three-neck flask, equipped with a condenser and a temperature controller, 492 µL of a 25 mM HAuCl4 aqueous solution (0.0123 mmol) diluted with 56 mL of milliQ water was heated with the heating plate. Once boiling appeared, under vigorous stirring, a mixture of 200 µL of sodium citrate (0.068 mmol, citrate/Au = 5.5) was injected. At *t* = 1 min, the solution turned light purple; at *t* = 5 min, it turned red, the heating was stopped, and the solution was cooled down to room temperature for half an hour. At this stage, the concentration of AuNPs is 0.66 × 10^−9^ mol/L. TEM images of the AuNPs are visible in Figure 1a, showing spherical nanoparticles. The lognormal distribution indicates a diameter of 21.7 nm with a narrow dispersion (σ = 0.15).

**UV-visible spectroscopy**. UV-visible spectra were recorded with a Jasco V730 (Jasco, Lisses, France) between 350 and 1000 nm with an acquisition time of 39 s for the saturation experiments and between 490 and 550 nm with an acquisition time of 18 s for the kinetics experiments. A quartz cuvette (QS high-precision cell 100-10-40) from Hellma Analytics (Hellma S.A.R.L., Paris, France) with a 10 mm light path was used. Baselines were systematically recorded with the cuvette filled with deionized water. Measurements of the spectra were performed without stirring. The exact LSPR position was evaluated by fitting the peaks with a Gaussian function restricted to a wavelength range of 30 nm centered on the maximum. Both the LSPR wavelength *λ*_LSPR_ and absorbance maximum were extracted. With this approach, the measurement accuracy for the peak wavelength was 0.05 nm.

**FTIR spectroscopy**. FTIR (Fourier Transform Infra-Red) spectra were recorded with a Bruker Tensor 27 spectrometer (Bruker France S.A.S, Wissembourg, France) in the transmission geometry with 70° incidence. Prior to the dropcastings, a reference spectrum was recorded by placing the clean silicon substrate in the sample holder of the spectrometer in exactly the same position as for the sample spectrum. Each FTIR spectrum is recorded with 4 cm^−1^ spectral resolution and results in the accumulation of 500 interferograms [41,42,43]. The spectra were recorded three times successively in order to let the nitrogen purge remove as much water vapor as possible in the sample compartment. Then, the ro-vibrational contributions of the water vapor were removed by signal processing to a level below 1 × 10^−4^ absorbance unit. Therefore, any feature greater than this value can be assigned to the vibration of a surface species.

**Saturation experiments** correspond to the progressive increase in thiol concentration until the saturation of the nanoparticle surface is detected (see discussion). Typically, the experiments consist of mixing *V*_1_ = 50 µL of variable concentration of a thiol solution with *V*_2_ = 2 mL of AuNPs solution (0.66 × 10^−9^ mol/L). The thiol solutions were obtained by diluting a mother solution in water. The mother solutions were fabricated with 2.5 mM of thiol (MUDA or MHDA) and 0.1 M of NaOH in water. The dissolution of thiols in water is facilitated by a slight increase in temperature. Then, the spectra were recorded 15 min after mixing to account for the functionalization kinetics. Each spectrum is obtained with a fresh mixture of AuNPs and thiol solution. Thiol concentrations were calculated so that their final concentrations in the cell (AuNPs + thiols) reached the following values: 0.13, 0.86, 2.07, 3.66, 4.91, 6.34, 17.68, and 39.01 µM.

**Kinetics experiments** allow monitoring the kinetics of nanoparticle functionalization for a given thiol concentration. The reaction was carried out in the spectrometer cuvette by mixing 2 mL of AuNPs at 0.66 × 10^−9^ mol/L with 50 µL of MUDA at 35 µM. The concentration of MUDA was 0.86 µM in the final solution. Spectrum acquisitions took 25 s, and they were recorded one after the other for 20 min. Stirring was stopped during this experiment.

**FTIR experiments** were used for assessing the nature of the molecules on the gold nanoparticles after surface saturation. Two samples were prepared using another AuNPs solution (18 nm, 1.22 × 10^−9^ mol/L): one with the as-prepared AuNPs solution (citrate-AuNPs) and a second one with AuNPs functionalized with MUDA (MUDA-AuNPs). For this latter, 0.75 mL of MUDA solution was added to 1 mL AuNPs solution so that the final concentration in MUDA was 2 µM, and the mixture was reacted for 15 min under stirring. In order to purify the AuNPs solutions and to remove unreacted molecules, AuNPs solutions were centrifugated for 30 min at 6000 rpm and recompleted with water or a mixture of 50 vol % ethanol in water after removal of the supernatant respectively for citrate-AuNPs and MUDA-AuNPs. The, these solutions were drop-casted on silicon substrates. Float-zone (FZ) silicon substrates were used because they contain an ultralow amount of inserted oxygen and allow monitoring the surface chemistry of AuNPs in the silicon oxide spectral region (1000–1400 cm^−1^). In these conditions, the symmetric and asymmetric carbonyl vibrations of the carboxylate can be detected, leading to distinguish citrate from MUDA on nanoparticles (see Results, below).

## 3. Results

### 3.1. FTIR Analysis of MUDA Functionalized AuNPs

**Figure 2:**
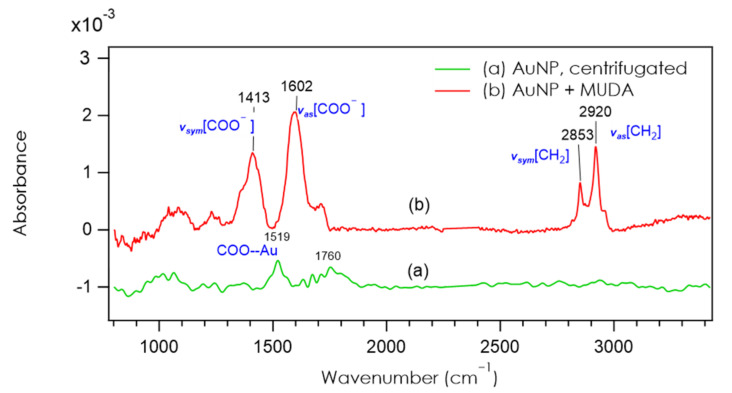
FTIR (Fourier Transform Infra-Red) absorption spectra of two AuNPs solutions, centrifugated and dropcasted on a silicon substrate, recorded in transmission with 70° incidence. Spectrum (**a**) corresponds to the Turkevich solution after centrifugation and spectrum (**b**) corresponds to the AuNP solution after functionalization with MUDA.

FTIR experiments were conducted with the pristine AuNP solution and with the AuNPs functionalized with 2 µM of MUDA. Both solutions were drop-casted on the silicon substrates. The two FTIR spectra are presented in Figure 2. Spectrum of Figure 2a of citrate-AuNPs shows a flat signal with only two features emerging from the noise. One peak is detected at 1519 cm^−1^, and other vibrations are visible around 1760 cm^−1^. The various features at 1760 cm^−1^ are remains of the stretching modes of water vapor that could not be totally suppressed. Due to the low amount of citrate, the noisy signal does not allow a clear identification of the peaks. Notice that for free citrate molecules, the asymmetric and the symmetric COO^−^ stretching modes are detected at 1385 cm^−1^ and 1575 cm^−1^ [27,44]. Here, the peak at 1519 cm^−1^ may be assigned to the COO moiety in interaction with the gold atoms. Figure 2b shows the spectrum of MUDA-AuNP, and unlike spectrum (a), the molecular vibrations are easily visible. The C–H stretching modes of the methylene group of MUDA at 2853 and 2920 cm^−1^ are clearly identified. Two strong modes at 1413 and 1602 cm^−1^ are also detected, and these are assigned to the carbonyl of the carboxylate moieties of MUDA. On both spectra, the small bump around 1100 cm^−1^ is due to a slight oxidation of the substrate. These FTIR spectra confirm the functionalization of AuNPs with MUDA. The next section will quantify this functionalization.

### 3.2. Surface Saturation Experiments

**Figure 3:**
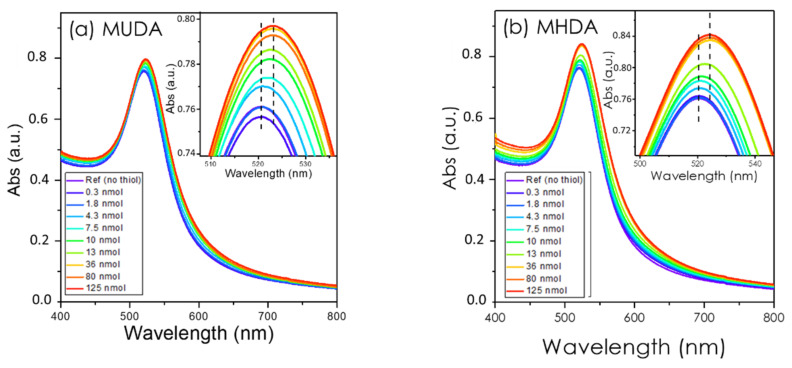
UV-visible spectra in solution of the progressive adsorption of (**a**) MUDA and (**b**) MHDA on 22 nm gold nanoparticles. The LSPR shift is 2.7 nm and 3.9 nm for MUDA and MHDA, respectively (see text for discussion). The insets show a zoom-in of the LSPR peak maximum.

Surface saturation experiments consist in mixing aliquots of previously prepared thiols solutions to a solution of AuNPs and recording a UV-visible spectrum 15 min after each addition. Figure 3 shows the spectral evolution for an increasing concentration of MUDA (Figure 3a) and MHDA (Figure 3b). They clearly show the LSPR absorbance peak measured at 520 nm for spherical gold nanoparticles in a pure water solvent [45,46]. The exact values of *λ*_LSPR_ are reported in Table 1.

The spectrum of Figure 3a shows that *λ*_LSPR_ progressively increases from 520.18 to 522.91 nm, corresponding to an experimental LSPR shift of $\Delta \lambda_{LSPR}=+2.7\;\mathrm{nm}$. This redshift is the clear indication that the optical index around the nanoparticle increased due to the progressive surface coverage of the gold nanoparticles by the MUDA molecules [38,47,48,49]. Although this qualitative behavior is well established, the quantitative exploitation has been less addressed so far and will be discussed in the next sections.

Similar experiments were carried out for the MHDA molecule, which differs from MUDA only by the length of the alkyl chain (16 vs. 11 –CH_2_– moieties for MHDA and MUDA, respectively). The UV-visible spectra measured for increasing concentrations of MHDA are shown in Figure 3b and Table 1. The LSPR peak position shifts from 520.18 to 524.07 nm ($\Delta \lambda_{\mathrm{LSPR}}=+3.89\;\mathrm{nm}$), due to the progressive coverage by the MHDA molecules.

The colloidal stability of citrate–AuNPs in water is ensured by the presence of charged citrate at the surface [50]. The preservation of colloidal stability by the progressive exchange of negatively charged citrate with MUDA or MHDA is possible thanks to the deprotonation of MUDA and MHDA molecules (pKa ≈ 5) in basic media (pH > 10). Indeed, the functionalization of AuNPs with neutral thiol molecules such as dodecanethiol (DDT), possessing a size close to the one of MUDA, leads to the decrease in the surface charge, inducing aggregation of the particles. In that case, a larger LSPR shift was detected (see Figure S1 $\Delta \lambda_{\mathrm{LSPR}}=+15.1\;\mathrm{nm}$), which has to be considered in addition to the functionalization [36,37,51].

**Figure 4:**
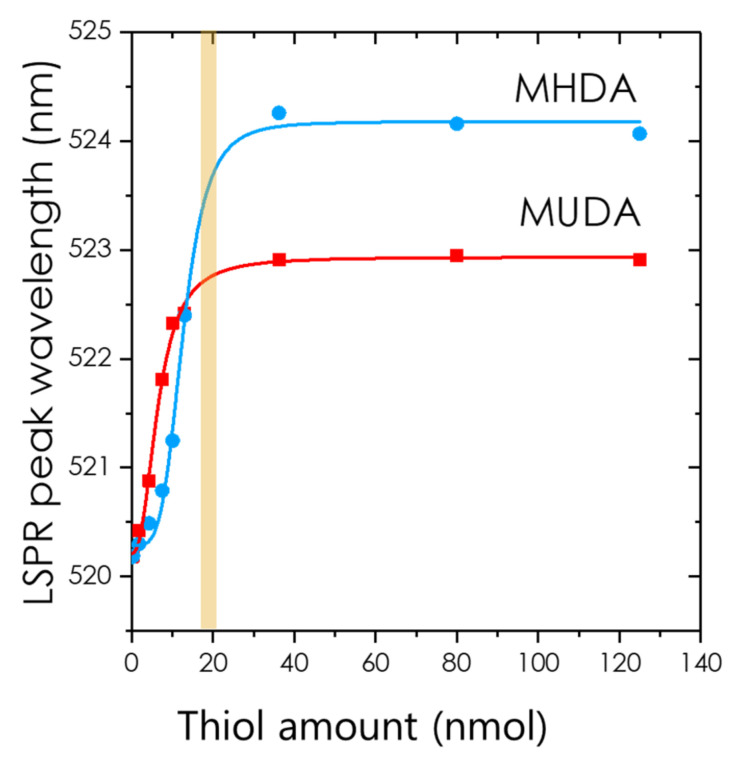
Evolution of the LSPR peak wavelength as a function of the added amount of thiol molecules in the case of MUDA (red square) and MHDA (blue circle). The curves are guides for the eye. The wide vertical line represents the transition threshold to the plateau and indicates that the full coverage of the nanoparticle is reached.

In the following, we have chosen to plot the LSPR peak wavelength as a function of the amount of thiol added to the initial AuNP solution (See Figure 4). Notice that instead of the LSPR shift, Van Duyne has explored the possibility of monitoring the evolution of intensity at the absorbance peak in the case of nano-triangles [52]. So did also Dahlin for spheres, but the sensitivity is achieved after heavy data processing [53,54]. Moreover, in some experiments, dilution processes or aggregation may blur the interpretation of the absorbance evolution compared to the wavelength monitoring. This is why we now focus on the LSPR wavelength shift.

### 3.3. Functionalization Reaction Kinetics

**Figure 5:**
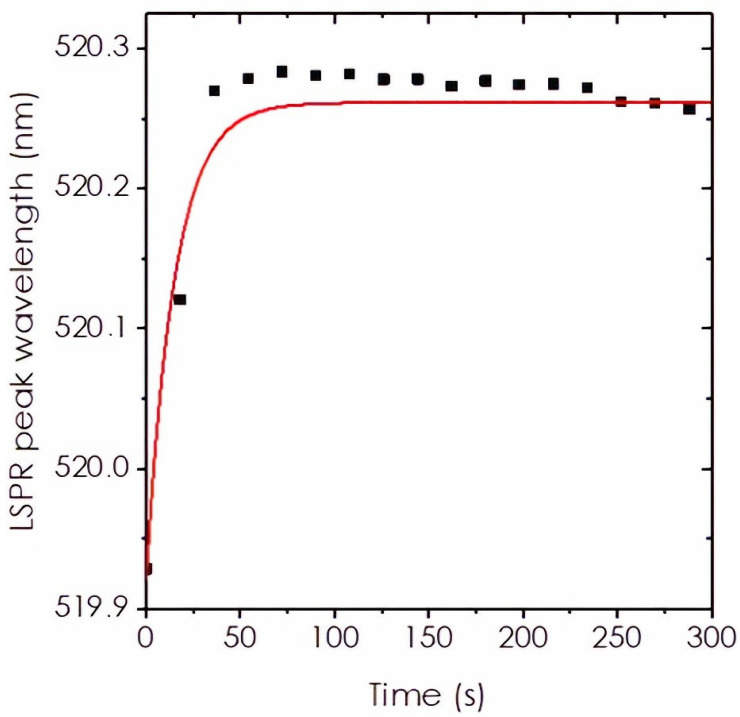
Evolution of the LSPR peak wavelength with time, providing information on the kinetics of functionalization of AuNPs with MUDA. Solution was not stirred during the acquisition. First, 50 µL of MUDA at 35 µM was added to 2 mL of AuNPs at 0.66 × 10^−9^ mol/L, leading to a final MUDA concentration of 0.86 µM in the reacting solution. The red line corresponds to a fit of the data with a single exponential.

Kinetics experiments were also carried out by mixing at *t* = 0 a solution of MUDA with AuNP solution. The progressive functionalization and its time evolution were monitored with the continuous acquisition of UV-visible spectra. The LSPR peak wavelengths were extracted from each spectrum and plotted versus time, as shown in Figure 5.

The global LSPR shift observed in this experiment is of 0.33 nm (519.93 to 520.26 nm), which is comparable with the shift observed in the saturation experiments at that concentration. This is coherent with the fact that the amount of MUDA added in this kinetic experiment is not sufficient to fully cover the nanoparticle surface. The plot from Figure 5 was fitted by a single exponential with a time constant τ=15 s. Therefore, the reaction completion can be estimated at $t_{reac}=3\times \tau =46\;s$. Without stirring, the reaction occurs by diffusion, which makes it slow enough to be monitored with our spectrometer. If the solution is stirred, the reaction occurs much faster, and we did not try to properly record this evolution.

### 3.4. Analytical and Theoretical Model for Predicting the LSPR Spectral Shift

**Figure 6:**
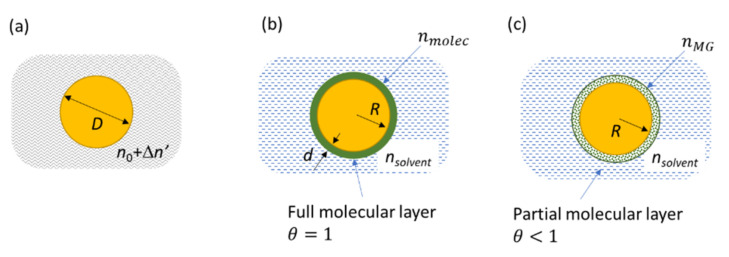
(**a**) A spherical nanoparticle of diameter *D* in a medium of index $n_{0}+\Delta n^{\prime }$ (**b**) nanoparticle functionalized by a molecular layer of thickness *d*, in a medium of index *n_solvent_*, (**c**) the same nanoparticle with a partial coverage θ<1. The refractive index of this partial monolayer $n_{MG}$ is calculated with the Maxwell–Garnett formula.

**Sensitivity factor of spherical nanoparticles** The dependence of the LSPR with the refractive index of its surrounding solvent, $n_{solvent},$ can be calculated using the electrostatic model or more accurately the Mie theory [45]. This situation is depicted in Figure 6a, and the spectra calculated with the Mie theory for increasing values for $n_{solvent}$ are shown in Figure S2 in the Supplementary Information (SI). If we restrain the variation of $n_{solvent}$ to the range 1.3–1.6 and write the new index $n_{solvent}=n_{0}+\Delta n^{\prime }$, the wavelength shift can be simply expressed with a linear relationship:

(1) $$\Delta \lambda =m\cdot \Delta n^{\prime }$$

where *m* is the sensitivity factor and depends on the size, on the shape, and on the nature of the nanoparticle. For a 22 nm spherical gold nanoparticle, $m=69\;\mathrm{nm}\cdot {\mathrm{RIU}}^{-1}$, where RIU stands for Refractive Index Unit (see Section S2 of the SI).

**Calculation of the LSPR shift for the full monolayer**. If we now consider a nanoparticle, initially in a solvent of index $n_{0}$, that is functionalized by a complete molecular monolayer characterized by an index $n_{molec}$, we cannot used Formula (1), since the molecular layer is thinner than the extension of the local electromagnetic field. This near field probes the molecular layer and the solvent over a distance *l_d_* called the electromagnetic decay length (see Figure S3 in the SI for a proper definition of *l_d_* and Figure S4 for its calculation). The molecular layer induces a small LSPR shift, and this effect has been first used by Englebienne for monitoring the affinity constants of biomolecules onto nanoparticles [38]. However, the proper relationship is due to the work of Van Duyne in the field of biosensors [47]. The resulting plasmon shift can be expressed as [48,55]:

(2) $$\Delta \lambda_{\mathrm{LSPR}}=m\cdot \Delta n\left[1-\exp\left(-\frac{2d}{l_{d}}\right)\right]$$

where *m* is the sensitivity factor and has the same value as in Equation (1), *d* is the thickness of the molecular layer, and *l_d_* is the electromagnetic decay length (see Figure 6b). However, Δn is now $n_{molec}-n_{solvent}$. More complex approaches were also proposed by Pollitt where the formulae are not linearized [56]. Sometimes, the plasmon shift is predicted by using an initial calibration procedure with liquids of known index of refraction [57]. An incorrect formula is proposed in Ref [58]. Therefore, we will draw the consequences from Equation (2) and calculate some numerical values for the wavelength shift.

The electromagnetic decay length is evaluated to be $l_{d}=0.45\times R$ for gold nanospheres (details of this evaluation are available in the Figure S4 in the SI). In the present case AuNPs have a radius R=11.0 nm; therefore, $l_{d}=4.95\;\mathrm{nm}$. We know that thiol molecules are linked to a surface with a tilt angle that is close to α=30° on flat gold substrates [59]. Therefore, the effective thickness *d* of a monolayer is smaller than the molecular length *l*, and d=l⋅cosα=1.32 nm (*l* = 1.52 nm) in Equation (2). The refractive index of a full monolayer is not easy to evaluate, since it depends on the nature of the molecule and its compactness on the surface. Here, we use $n_{\mathrm{molec}}=1.46$ according to the value measured for a compact DDT monolayer by Goldmann [60] and from similar values published by Messersmith [58]. Therefore, the solvent was pure water $\Delta n=n_{molec}-n_{solvent}=1.46-1.33=0.13$. Now that every parameter is known, it is possible to use Formula (2), and we obtain $\Delta \lambda_{\mathrm{LSPR}}=3.70\;\mathrm{nm}$ for MUDA. For MHDA, using the same refractive index for the full monolayer as previously ($n_{molec}=1.46$), and given that the molecule length with a tilt of 30° is now 1.84 nm (*l* = 2.130 nm), Formula (2) yields $\Delta \lambda_{\mathrm{LSPR}}=4.71\;\mathrm{nm}$. These calculated values are close to the measured ones and will be discussed in the following.

**Partial coverage of the nanoparticle: the Maxwell–Garnett approach.** Here, we address the case when the molecules do not fully cover the nanoparticles as shown in (see Figure 6c). This partial coverage corresponds to cases of low molar concentrations of thiol derivatives or to the intermediate situations during kinetic experiments such as in Figure 5, where molecules progressively cover the nanoparticle surface. One question is to unravel how the monitoring of the wavelength shift could give access to the evolution of the coverage with time. In other words, is $\Delta \lambda_{\mathrm{LSPR}}$ linearly related to the coverage θ? A partial coverage corresponds to a medium where the low density of the molecule leaves room for some solvent molecules. The resulting refracting index can be calculated using the Effective Medium Approach and namely the Maxwell–Garnett formula (MG) [46,61]. MG yields the dielectric permittivity $\varepsilon_{MG}$ of a mixed medium (solvent $\varepsilon_{solvent}$) containing some inclusions of another material (here, the adsorbed molecules, $\varepsilon_{molec}$) dispersed with a volumic fraction *f*. In the present case, the volume fraction of adsorbate is equivalent to the surface coverage θ. θ varies between 0 and 1, and a full monolayer corresponds to θ=1. Therefore, $\varepsilon_{MG}$ writes:

(3) $$\varepsilon_{MG}=\varepsilon_{solvent}\frac{\varepsilon_{molec}\left(1+2\theta \right)+2\varepsilon_{solvent}\left(1-\theta \right)}{\varepsilon_{molec}\left(1-\theta \right)+\varepsilon_{solvent}\left(2+\theta \right)}.$$

This expression simplifies into:

(4) $$\varepsilon_{MG}=\varepsilon_{solvent}\frac{1+2q\theta }{1-q\theta }\;\mathrm{with}\;q=\frac{\varepsilon_{molec}-\varepsilon_{solvent}}{\varepsilon_{molec}+2\varepsilon_{solvent}}.$$

If $\varepsilon_{molec}$ is close to $\varepsilon_{solvent}$, then q≪1, and Equation (4) can be linearized. In the present case, $\varepsilon_{solvent}=1.33^{2}=1.77$ and $\varepsilon_{molec}=1.46^{2}=2.13$; therefore, q=0.064, which satisfies q≪1. The linearization is reasonable, and we obtain the equivalent refractive index of the partially complete molecular layer $n_{MG}$ as:

(5) $$n_{MG}={\left(\varepsilon_{MG}\right)}^{1/2}=n_{solvent}\left(1+\frac{3}{2}q\theta \right).$$

This result is crucial because it simply states that in the case of a mixture of materials with indices close to each other, the resulting index is simply proportional to the surface coverage θ.

**Application to the case of plasmonic nanoparticles.** Let us now wrap up Equation (5) with Equation (2). Now, $\Delta n=n_{MG}-n_{solvent}$ and can be written with Equation (5): $\Delta n=\frac{3}{2}\;q\cdot \theta \cdot n_{solvent}$. Then,

(6) $$\Delta \lambda_{LSPR}=m\cdot \frac{3}{2}\;q\cdot \theta \cdot n_{solvent}\left[1-\exp\left(-\frac{2d}{l_{d}}\right)\right].$$

Every parameter of Equation (6) was calculated; therefore, in our conditions,

(7) $$\Delta \lambda_{LSPR}=3.63\times \theta \;(\mathrm{nm})\;\mathrm{for}\;\mathrm{MUDA}\;$$

(8) $$\Delta \lambda_{LSPR}=4.63\times \theta \;(\mathrm{nm})\;\mathrm{for}\;\mathrm{MHDA}.$$

Equations are valid for 22 nm gold spherical nanoparticles in water with a partial coverage, and the results are summarized in Table 2.

## 4. Discussion

### 4.1. AuNP Full Functionalization—Geometrical Calculation and Experimental Determination

The existence of the plateau in the adsorption of MUDA and MHDA shown in Figure 4 suggests that a full nanoparticle coverage is reached at around 20 nmol for both molecules of this study. Some simple geometrical considerations could confirm this value. Let us start by calculating the AuNP concentration in solution, assuming that every HAuCl_4_ species was reduced into metallic gold. If we suppose that the nanoparticles are crystallized into an *fcc* lattice and are spherical (diameter *D*), the number of gold atoms they contain is given by $N_{at}=\frac{\pi }{6}.\frac{\rho D^{3}}{M}.N_{Av}$ with $\rho =19.3\;{g/\mathrm{cm}}^{3}$ and *M* = 197 g/mol being respectively the volume weight and the molar weight of gold, and *N_Av_* being the Avogadro number [62]. For gold, this formula simply writes $N_{at}=30.9\cdot D^{3}$ where *D* is in nm. Therefore, the nanoparticle concentration can be evaluated with $C_{NP}=\left[{\mathrm{HAuCl}}_{4}\right]/N_{at}$ where $\left[{\mathrm{HAuCl}}_{4}\right]$ is the concentration of chloroauric acid in the final volume (after addition of citrate). It writes (*D* being in nm):

(9) $$C_{NP}=\frac{\left[{\mathrm{HAuCl}}_{4}\right]}{30.9\times D^{3}}.$$

In the present case with 22 nm AuNP, $C_{NP}=0.66{\times 10}^{-9}\;\mathrm{mol}/L$.

Let *N*_thiol_ be the amount of thiol molecules that a nanoparticle can accommodate after the formation of a full monolayer. *N*_thiol_ is proportional to its surface $S_{NP}=\pi D^{2}$ and depends on the average thiol surface density *p* within an SAM (number of thiol molecules per nm²). It writes: $N_{thiol}=\pi D^{2}p.$ From Ref. [59], $p=1/\left(0.2165{\;\mathrm{nm}}^{2}\right)=4.619\;\mathrm{molec}/{\mathrm{nm}}^{2}$ for flat Au(111). More realistic values have been determined for spherical nanoparticles ranging from 5.26 to $5.88\;\mathrm{molec}/{\mathrm{nm}}^{2}$ [63,64,65]. In the following, a value of $p=5.5\;\mathrm{molec}/{\mathrm{nm}}^{2}$. Subsequently, for 22 nm AuNP, we calculate that $N_{thiol}=8440$ thiol molecules are necessary for saturating the nanoparticle surface. It leads to a concentration of thiol of $C_{thiol}^{sat}=C_{NP}\times N_{thiol}$. For gold, this yields the following two simple and equivalent formula (*D* being the nanoparticle diameter in nm):

(10) $$C_{thiol}^{sat}=17.4\times C_{NP}\times D^{2}$$

(11) $$C_{thiol}^{sat}=0.56\times \frac{\left[HAuCl_{4}\right]}{D}.$$

In order to compare with our experimental results, it is easier to calculate the absolute quantity of thiol molecules needed to functionalize the AuNP contained in the initial suspension (volume *V*_2_ = 2 mL) used in the experiments. This number is simply $C_{NP}\times V_{2}\times N_{thiol}$ = 11.1 nmol. In our experiments, we obtained a larger value, 20 nmol (see Figure 4), which corresponds to *N_thiol_* = 15,160 and to a ligand packing density of 10 molecules per nm^2^. From the data of Table 1, this saturation quantity can be converted into the saturation concentration. When 20 ± 2 nmol was poured into the AuNPs solution, the total volume was 2.05 mL. Therefore, the thiol concentration in the cuvette was $C_{thiol}^{exp}=10\;\pm 1\;µM$, and the NP concentration, $C_{NP}=0.64\times 10^{-9}\;M$. Equation (10) yields $C_{thiol}^{sat}=5.39\;µM$. The clear plateau for the two curves of Figure 4 shows that MUDA and MHDA no longer adsorb when the threshold of 20 ± 2 nmol has been reached. The larger amount of thiol necessary to reach saturation evidenced by the experiments points to other chemical and physical phenomena that are not been considered: the formation of a multilayer at the nanoparticle surface, larger reactive surface of the nanoparticle, and partial oxidation of the thiol molecules that prevent them from reacting with gold. Discussing in details these phenomena is out of our scope. We can only rule out the formation of thiol multiple layers, since it would greatly affect the LSPR shift, due to a higher thickness of the layer, which is not observed experimentally. In any case, our Formulas (10) and (11) provide the correct order of magnitude and establish the minimum of thiol amount to be added in a nanoparticle suspension for reaching surface saturation.

### 4.2. LSPR Shift after Full Molecular Functionalization

The LSPR shift observed after full nanoparticles functionalization is 2.7 nm and 3.9 nm for MUDA and MHDA, respectively. This is slightly smaller than the calculated values of 3.70 and 4.71 nm, respectively (see Table 1). However, notice that the model is based on a set of assumptions considering a compact molecular layer, with molecules tilted by 30° to the normal of the surface, and with an optical index of $n_{molec}=1.46$. It is impossible to confirm experimentally the accuracy of these assumptions. For example, taking an angle of 50° as reported in some articles can lead to an LSPR shift of 2.9 and 3.8 nm, respectively, for MUDA and MHDA, which offers a better agreement with our results [66]. In the SI, the influence of slight variations of these input parameters are discussed (see Section S4 of the SI). Our model is especially interesting for understanding the trends in LSPR wavelength variations, when for example the molecular lengths is increased, when the nanoparticle diameter is modified, or when the solvent is changed.

### 4.3. Kinetics and Time for Completing Nanoparticle Functionalization

Figure 5 shows the mixing of 22 nm AuNPs with MUDA with a total volume of 2.05 mL, so that the concentrations of AuNPs and MUDA are $0.64\times 10^{-9}\;\mathrm{mol}/L$ and 0.008 µM, respectively. It results in a progressive shift of the LSPR peak that follows an exponential evolution with a time constant τ=15 s. From the demonstration summarized by Equations (6)–(8), we know that the peak wavelength shift is proportional to the coverage. Therefore, we can affirm that the coverage also follows an exponential law with time. This exponential evolution suggests that the reaction follows the classical Langmuir kinetics [67,68]. Within the Langmuirian model, the rate constant k=1/τ is a combination of the intrinsic rate constant of adsorption $k_{a}$ and the intrinsic rate constant of desorption $k_{d}$. It writes $k=k_{a}\cdot C_{thiol}+k_{d}$. Here, we neglect the desorption of thiols; therefore, the rate constant k is simply proportional to the thiol concentration: $k\approx k_{a}\cdot C_{thiol}$. In our experiments, we have measured that the completion of the reaction is reached after $t_{sat}=3\times \tau =45\;s$ when $C_{thiol}=0.86\;µM$. These numbers are in sharp contrast with other studies where the reaction time is often set “overnight” [44,69]. Actually, our approach provides with an approximation of the evolution time, since many factors affect these kinetics. The first one is that when $C_{thiol}>0.86\;µM$, the adsorption will saturate the nanoparticle surface, and the time evolution will be stopped. Therefore, our value of $t_{sat}$ is an upper limit for the completion time. Moreover, the mechanism of thiol adsorption is definitively more complex than just a simple Langmuir kinetics [67]. MUDA adsorption kinetics on a flat gold surface was studied in an earlier study, which confirms that there are two kinetic steps (a first quick step, then a quick one) depending on the thiol concentration [70]. Therefore, our approach provides a very useful method that enables evaluating the best conditions in terms of thiol concentration and immersion time for functionalizing gold nanoparticles.

## 5. Conclusions

Chemical functionalization with two thiol derivatives (MUDA, MHDA) of 22 nm spherical gold nanoparticles (AuNPs) in solution was studied with UV-visible spectroscopy by monitoring the wavelength of the LSPR peak. Our study focused on three aspects of these families of reaction. First, upon adsorption, the LSPR peak increases from 520.2 to 522.9 nm or 524.1 nm for MUDA and MHDA, respectively, and it reached a plateau even when more concentrated thiols solutions were added. This plateau corresponds to the surface saturation of the AuNPs (concentration $0.66\times 10^{-9}\;\mathrm{mol}/L$), which is measured for a thiol concentration of 10 µM. It corresponds to 15,000 molecules per AuNP. We have shown that geometrical calculations for 22 nm spherical nanoparticles predict a lower amount of molecules (8400 molecules). This geometrical calculation can be used as a lower limit when designing an experiment where the goal is to fully functionalize the nanoparticle surface. A second aspect of our study dealt with measuring and predicting the LSPR peak shift for different molecules. Our experiments showed that the experimental values of $\Delta \lambda_{LSPR}$ were 2.7 nm (MUDA) and 3.9 nm (MHDA). Our analytical model agrees with these values. Finally, the third aspect of our work is related to the kinetics of the functionalization reaction. It follows approximately a Langmuir kinetic, and with a thiol concentration of 0.86 µM, the reaction completion is attained within less than 45 s, which is much faster compared to the typical time used in the literature for functionalization.

This study will provide a set of useful methods and sound orders of magnitude for properly handling the thiol chemistry on gold nanoparticles. It can be applied to other thiol derivatives provided they did not trigger nanoparticle aggregation.

## Figures and Tables

**Table 1:** Experimental parameters for the saturation experiment with MUDA and MHDA (*V*_1_ = 50 µL) and measured LSPR peak wavelength.

#	[Thiol] in the Added Aliquot (µM)	*n*_thiol_ Added (nmol)	[Thiol] in the Solution (µM)	*λ*_LSPR_ (nm) for MUDA (Measured)	*λ*_LSPR_ (nm) for MHDA (Measured)
0	0	0	0	520.18	520.18
1	6	0.28	0.13	520.19	520.19
2	35	1.75	0.86	520.42	520.30
3	85	4.25	2.07	520.88	520.49
4	150	7.50	3.66	521.81	520.79
5	201	10.06	4.91	522.33	521.25
6	260	13.00	6.34	522.42	522.40
7	725	36.25	17.68	522.91	524.26
8	1599	79.97	39.01	522.95	524.16
9	2500	125.00	60.98	522.91	524.07

**Table 2:** Calculated and measured LSPR wavelength shifts for the two molecules of the present study. The molecules length is given assuming a tilt angle from the normal to the surface of α=30°.

Molecules	Length (*d*)/nm	*n*_solvent_	$\Delta \lambda_{LSPR}$ Calc. Satur/nm	$\Delta \lambda_{LSPR}$ Calc. Partial Cov/nm	$\Delta \lambda_{LSPR}$ Meas. /nm
**MUDA**	1.32	1.33	3.70	3.63×θ	2.7
**MHDA**	1.84	1.33	4.71	4.63×θ	3.9

## Supplementary Materials

The following supporting information can be downloaded at: https://www.mdpi.com/article/10.3390/nano12020292/s1, Figure S1: UV-visible spectra corresponding to DDT adsorption on AuNPs in solution. Initial gold nanoparticle concentration was 1.22 × 10^−9^ M. Experiments are carried out under stirring and this sometimes induces the oscillations observed in the spectra, Figure S2: (a) Evolution of the extinction cross section of 22 nm AuNP in a medium whose index is increased from 1 to 1.8. Calculations are done with the Mie theory. (b) Plot of the extinction maximum (solid red lines, left axis) as a function of the optical index of the surrounding medium. Values of the max extinction cross section (blue open circles, right axis). Within the range of 1.3–1.6, the evolution is approximately linear with a slope of *m* = 69 nm·RIU^−1^, Figure S3: (a) Spherical nanoparticle, functionalized by a molecular layer of thickness d, in a medium of index *n*_0_; The near field radiated by the particle extends over a length *l*_d_. (b) 2D representation of the near-field enhancement by a gold nanoparticle in water, when illuminated at its plasmon resonance (*λ* = 520 nm). The intensity of electric field is enhanced of a factor of 20 in the vicinity of the nanoparticle surface and decay as 1/*r*^3^, Figure S4: Decay of the radiated electric field (near field) along the axis xx’ and zz’ for a gold nanoparticle in water, Table S1: Experimental parameters for the saturation experiment with DDT and measured LSPR peak (successive thiol additions with *V*_1_ = 187.5 µL), Table S2: Values of the lambda max as a function of the index of the surrounding solvent.
