# Supplementary material for: How to Use Localized Surface Plasmon for Monitoring the Adsorption of Thiol Molecules on Gold Nanoparticles?
Source: Nanomaterials (Basel). 2022 Jan 17;12(2):292. doi: 10.3390/nano12020292 (PMC8778005; doi:10.3390/nano12020292)
Supplement: Supplementary file 1 [file nanomaterials-12-00292-s001.zip › nanomaterials-1541222 - Supplementary Materials v1.pdf]

nanomaterials

Supporting Information

How to Use Localized Surface Plasmon for Monitoring the Adsorption of Thiol Molecules on Gold Nanoparticles?

Angeline S. Dileseigres, Yoann Prado and Olivier Pluchery *

CNRS, Institut des Nanosciences de Paris (INSP) UMR7588, Sorbonne Université, 4 place Jussieu, 75005 Paris, France; angeline.dileseigres@insp.jussieu.fr (A.S.D.); yoann.prado@insp.jussieu.fr (Y.P.)
* Correspondence: olivier.pluchery@insp.jussieu.fr; Tel.: +33 1 44 27 94 10

S1. Progressive Nanoparticle Surface Saturation with DDT

Another AuNPs solution (18 nm, 1.22 × 10^{-9} mol/L) has been used here. The saturation experiments were carried out with DDT with successive V_1 = 187.5 µL addition of the alkylthiols in a mixture of ethanol / water.

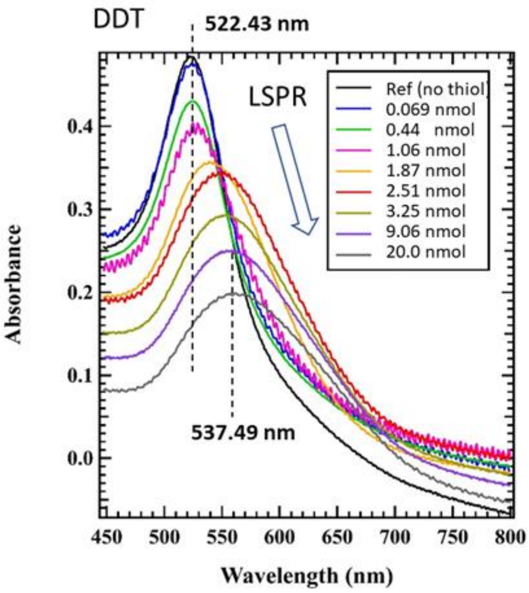

Figure S1. UV-visible spectra corresponding to DDT adsorption on AuNPs in solution. Initial gold nanoparticle concentration was 1.22 × 10^{-9} M. Experiments are carried out under stirring and this sometimes induces the oscillations observed in the spectra.

Table S1. Experimental parameters for the saturation experiment with DDT and measured LSPR peak (successive thiol additions with V_1 = 187.5 µL).

#	[Thiol] in the Added Aliquot (µM)	n_{thiol} added (nmol)	V_{sol} (µL)	[Thiol] in the Solution (µM)	Vol % of EtOH in Solvent	λ_{LSPR} (nm) for DDT (Measured)	Solvent Refractive Index
0	0	0	1000	0	0%	522.70	1.333
1	0.367	0.069	1188	0.058	16%	523.58	1.344
2	1.97	0.44	1376	0.32	27%	524.07	1.352
3	3.33	1.06	1564	0.68	36%	527.10	1.357
4	4.33	1.87	1752	1.07	43%	540.64	1.359
5	3.41	2.51	1940	1.3	48%	548.84	1.361
6	3.92	3.25	2128	1.53	53%	552.15	1.362
7	31	9.06	2316	3.92	57%	555.01	1.363
8	58.3	20.0	2504	8	60%	557.54	1.364

S2. Calculation of the λ_{max} for Increasing Solvent Indices with the Mie Theory

The Mie theory was used, and the calculations are those from the Bichromatics calculator [1,6]. This allows plotting the wavelength of the maximum of the extinction cross section as a function of the refractive index of the solvent.

The evolution of the LSPR peaks as a function of $n_{solvent}$ can be seen in Figure S2a. Figure S2b shows that this evolution is not linear but can be approximated to a straight line in the range of n = 1.3–1.6 with a slope of 69 nm/RIU. This slope is the sensitivity factor m used in the Equations (1), (2) and (6) of the main text. If we write the new index $\mathrm{n_{solvent}} = \mathrm{n_0} + \Delta\mathrm{n}'$, the wavelength shift can be simply expressed with a linear relationship:

$$\Delta\lambda = m \cdot \Delta n' \quad \text{(S1)}$$

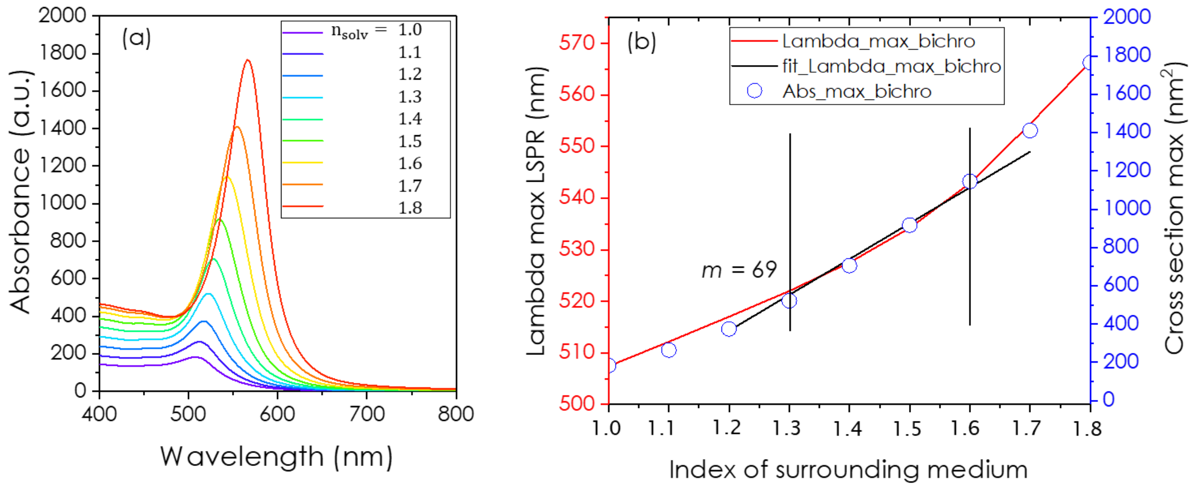

Figure S2. (**a**) Evolution of the extinction cross section of 22 nm AuNP in a medium whose index is increased from 1 to 1.8. Calculations are done with the Mie theory. (**b**) Plot of the extinction maximum (solid red lines, left axis) as a function of the optical index of the surrounding solvent. Value of the max extinction cross section (blue open circles, right axis). Within the range of 1.3–1.6, the evolution is approximately linear with a slope of m = 69 nm·RIU^{-1}.

Table S2. Values of the lambda max as a function of the index of the surrounding solvent.

Index (RIU)	Lambda Max (nm)	Extinction Max (nm²)
1	507.42	183.26
1.1	512.17	264.38
1.2	517.03	374.80
1.3	522.02	521.45
1.4	527.53	705.21
1.5	534.22	916.80
1.6	542.93	1145.12
1.7	554.42	1410.65
1.8	566.43	1766.00

S3. The Electromagnetic Decay Length for a Nanosphere

A sphere of radius R excited by the electromagnetic field E_0, radiates an optical near field that decays away from the nanoparticle as sketched in Figure S3.

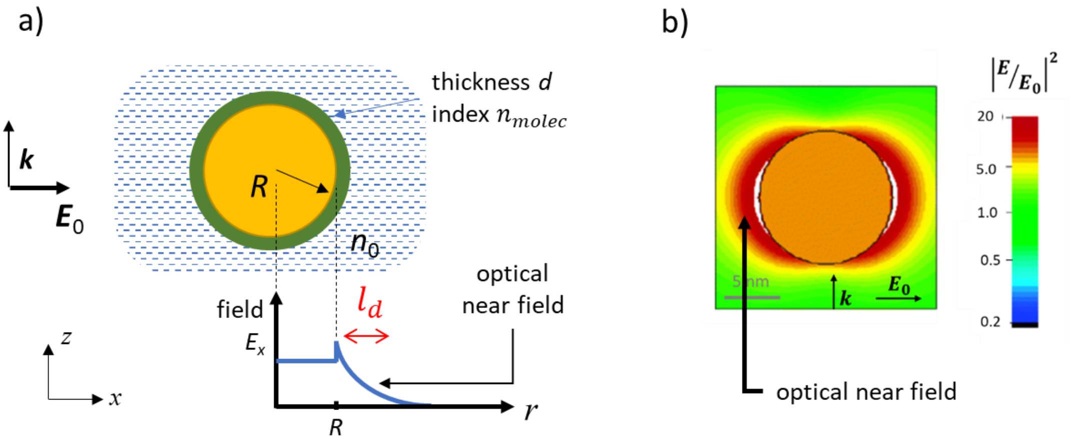

Figure S3. (**a**) Spherical nanoparticle, functionalized by a molecular layer of thickness d, in a medium of index n_0; The near field radiated by the particle extends over a length l_d. (**b**) 2D representation of the near-field enhancement by a gold nanoparticle in water, when illuminated at its plasmon resonance (λ = 520 nm). The intensity of electric field is enhanced of a factor of 20 in the vicinity of the nanoparticle surface and decay as $1/r^3$. [reproduced after Ref [3]].

Implicitly, a decay length l_d is related to an exponential decay, where the electric field would be of the form:

$$E_x = E_0 + A\, exp\left(-\frac{r-R}{l_d}\right) \quad \text{(S2)}$$

However, the expression of the near field exhibits a rapid decrease in $1/r^3$ and not an exponential decay. The near field radiated by a metallic sphere along the xx′ and zz′ directions, under an excitation $E_0 \boldsymbol{u_x}$ is given by [2,5]:

$$E_{xx\prime} = E_0\left(1 + 2\frac{\alpha\prime}{x^3}\right) \text{ and } E_{zz\prime} = E_0\left(1 - \frac{\alpha\prime}{x^3}\right) \quad \text{(S3)}$$

where $x = r/R$ and α' is the reduced polarizability of the particle. A representation of this near field is given in Figure S3b.

Nevertheless, it is possible to fit the Equation (S3) with an exponential decay such as the one is Equation (S2) as shown in Figure S4. At the resonance, and for a gold nanoparticle, the reduced polarizability is $\alpha' = 2.63$ and the fit yields for$E_{xx'}$ and $E_{zz'}$ of Equation (S3):

$$E_{xx'} = 1.04 \times E_0 + 4.83\ exp\left(-\frac{x-1}{0.45}\right)$$

$$E_{zz'} = 0.98 \times E_0 - 2.42\ exp\left(-\frac{x-1}{0.45}\right)$$

This means that:

$$l_d = 0.45 \times R \tag{S4}$$

In the present case AuNP have a radius $R = 11.0$ nm, therefore, $l_d = 4.95$ nm.

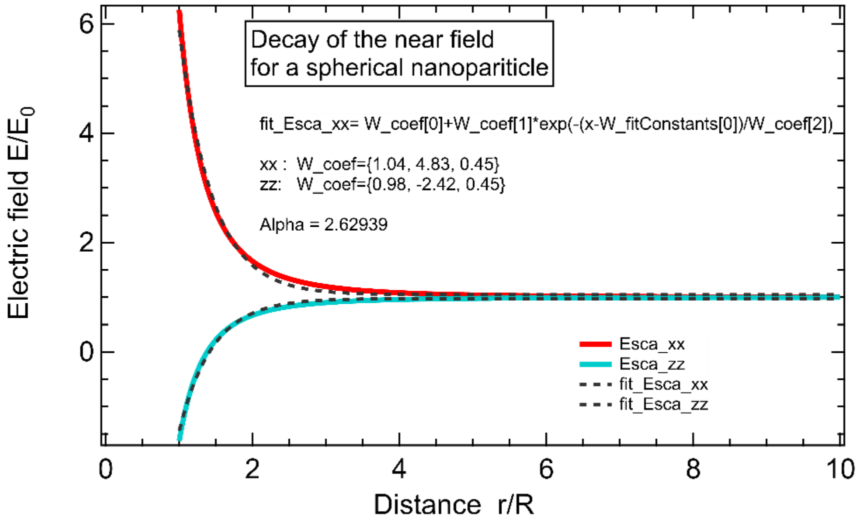

Figure 4. Decay of the radiated electric field (near field) along the axis xx′ and zz′ for a gold nanoparticle in water.

S4. Influence of the Uncertainty of the Parameters on the Calculation of the LSPR Wavelength Shift.

As discussed in the main text, the LSPR shift observed after full nanoparticles functionalization is 2.7 nm and 3.9 nm for MUDA and MHDA respectively which differ from the calculated values of 3.7 and 4.7 nm respectively (see Figure 3 of the main text). However, notice that the model is based on a set of three assumptions considering 1) an ideal molecular layer like the one sketched in Figure 6b: a molecular layer whose optical index is $n_{molec} = 1.54$, 2) with molecules standing perpendicularly to the nanoparticle surface and 3) forming a compact monolayer. None of these three assumptions are completely accurate and implies small deviation for the values of $\Delta\lambda_{LSPR}$.

For example, if instead of $n_{molec} = 1.54$, one sets $n_{molec} = 1.52$, the $\Delta\lambda_{LSPR}$ calculated with Equation (2) of the main text goes from 6.65 to 6.02 nm.

Second, if the thiol molecules are considered tilted of 30° like they are on flat Au(111) surfaces [4], the effective monolayer thickness is reduced from 1.520 nm to 1.316 nm and subsequently $\Delta\lambda_{LSPR}$ decreases from 6.65 to 5.97 nm. This effect might be even stronger with the long MHDA molecules where this is highly unlikely that the 16-carbon long chain stands straight away from the surface.

Third, the molecular density on the nanoparticle surface might also be reduced compared to a flat well-ordered gold surface. This will also minimize $\Delta\lambda_{LSPR}$.

Finally, the model of Equation (2) correctly captures the trend for $\Delta\lambda_{LSPR}$ but its weak point lies in the precision of the input parameters. This model will be therefore especially efficient for comparing molecules with each others.

References

1. Laven P. MiePlot 4.3.05. Calculation of Mie scattering from a sphere. Available online: http://www.philiplaven.com/mieplot.htm (accessed on 20 December 2021).
2. Novotny L.; Hecht B. *Principles of Nano-Optics*, 2nd ed.; Cambridge University Press: Cambridge, UK, 2012.
3. Rodríguez-Fernández, J.; Pérez-Juste, J.; García de Abajo, F.J.; Liz-Marzán L.M. Seeded Growth of Submicron Au Colloids with Quadrupole Plasmon Resonance Modes. *Langmuir* **2006**, *22*, 7007–7010.
4. Vericat, C.; Vela, M.E.; Salvarezza, R.C. Self-assembled monolayers of alkanethiols on Au(111): surface structures, defects and dynamics. *Phys. Chem. Chem. Phys.* **2005**, *7*, 3258–3268.
5. Watkins, W.L.; Assaf, A.; Prévot, G.; Borensztein, Y. Dichroic Plasmonic Films Based on Anisotropic Au Nanoparticles for Enhanced Sensitivity and Figure of Merit Sensing. *J. Phys. Chem. C* **2021**, *125*, 11799–11812
6. Pluchery, O.; Watkins, W.L. Bichromatics, spectrum calculator. Available online: https://www.bichromatics.com/calculator/ (accessed on 20 December 2021).
